# Upregulation of DNA Sensors in B16.F10 Melanoma Spheroid Cells After Electrotransfer of pDNA

**DOI:** 10.1177/1533033818780088

**Published:** 2018-06-07

**Authors:** Katarina Znidar, Masa Bosnjak, Tanja Jesenko, Loree C. Heller, Maja Cemazar

**Affiliations:** 1 Faculty of Health Sciences, University of Primorska, Koper, Slovenia; 2 Department of Experimental Oncology, Institute of Oncology Ljubljana, Ljubljana, Slovenia; 3 Frank Reidy Research Center of Bioelectrics, Old Dominion University, Norfolk, VA, USA; 4 School of Medical Diagnostic and Translational Sciences, College of Health Sciences, Old Dominion, University, Norfolk, VA, USA

**Keywords:** B16.F10 melanoma, spheroids, DNA sensors, interferon β, tumor necrosis factor α, electrotransfer of pDNA

## Abstract

Increased expression of cytosolic DNA sensors, a category of pattern recognition receptor, after control plasmid DNA electrotransfer was observed in our previous studies on B16.F10 murine melanoma cells. This expression was correlated with the upregulation of proinflammatory cytokines and chemokines and was associated with cell death. Here, we expanded our research to include the influence of features of cells in a 3-dimensional environment, which better represents the tumors’ organization *in vivo*. Our results show that lower number of cells were transfected in spheroids compared to 2-dimensional cultures, that growth was delayed after electroporation alone or after electrotransfer of plasmid DNA, and that DNA sensors DDX60, DAI/ZBP1, and p204 were upregulated 4 hours and 24 hours after electrotransfer of plasmid DNA. Moreover, the cytokines interferon β and tumor necrosis factor α were also upregulated but only 4 hours after electrotransfer of plasmid DNA. Thus, our results confirm the results obtained in 2-dimensional cell cultures demonstrating that electrotransfer of plasmid DNA to tumor cells in spheroids also upregulated cytosolic DNA sensors and cytokines.

## Introduction

Gene therapy is gaining its importance in clinical applications as a replacement of missing or nonfunctional proteins, in the enhancement of the immune response against cancer, and in other applications.^1–4^ There have been approximately 80 clinical studies for the treatment of different diseases utilizing the gene delivery method electroporation or electrotransfer (http://ClinicalTrials.gov). Gene electrotransfer is a method by which cells are exposed to external electric field in order to increase the permeability of the cell membrane and thus enable the entry of plasmid DNA (pDNA) into the cell. To better understand the complexity of gene electrotransfer process, preclinical *in vitro* and *in vivo* studies are performed exploring underlying mechanisms.^5–9^

In preclinical cancer studies using gene electrotransfer, pDNA devoid of a therapeutic gene serves as a control for therapeutic pDNA. This DNA causes tumor growth delay, increased survival time, and complete tumor regression.^8,10–16^ A possible explanation for tumor growth delay and regression is the activation of cytosolic DNA sensors.^11,13,14,17^ After electrotransfer, pDNA can enter the cells via endocytosis or hypothetically through pores directly to the cytosol.^18,19^ The endosomal DNA is released to the cytosol in order to reach the cell nucleus. Thus, the result of electrotransfer is the presence of pDNA inside the cytosol, which is, in normal cells, DNA free.^20^ Free DNA in cytosol may bind to cytosolic DNA sensors, which are a subgroup of pattern recognition receptors, and consequently activate different signaling pathways that further lead to the activation of immune response or cell death.^21–23^

In a previous study, we demonstrated the upregulation of specific cytosolic DNA sensors after electrotransfer of pDNA in melanoma cells in culture.^11^ This upregulation was accompanied with increased expression of cytokine interferon β (IFN-β). We expanded this research to different tumor cell types, where we also observed upregulation of some DNA sensors in mammary adenocarcinoma (TS/A) and fibrosarcoma (WEHI 164) accompanied with increased expression of IFN-β and TNF-α.^17^ We also demonstrated a possible autocrine and/or paracrine pathway through the interferon α/β receptor 1 and tumor necrosis factor receptor 1 (TNFR-1) receptors.

Although we obtained confirming results in different tumor cell lines, here we tested the 3-D conformation of cells for a better understanding and prediction of the outcome *in vivo.* Two-dimensional monolayer cell culture cannot reproduce the complex organization and architecture of tissue. Consequently, numerous signals that govern different cellular processes are lost when cells are grown in 2-dimensional (2-D) plastic substrate. Three-dimensional (3-D) cell cultures also possess several *in vivo* features of tumors such as cell–cell interactions, hypoxia, limited drug penetration, and the production of extracellular matrix.^24^ Specifically, for tumor spheroids, the hypoxic center of the spheroid and lack of nutrients due to impaired diffusion mimic the *in vivo* situation. As such they represent a bridge from 2-D cell cultures to *in vivo* tumor model experiments.

For this study, we chose a pulse protocol that has been tested on B16.F10 cells in suspension, on B16.F10 tumors *in vivo*, clinically for the delivery of chemotherapeutic agents and is currently in gene therapy clinical trials. Delivery of these pulses kills approximately 10% of B16.F10 cells in suspension.^11,25^ No antitumor effects are observed after delivering these pulses to B16.F10 melanoma tumors.^26^ This pulse regimen has been tested in humans, and no antitumor effect was observed histologically.^27^ Eight of these pulses are used clinically for the delivery of chemotherapeutic agents^28^ and 6 of these pulses are in clinical trials for plasmid delivery^29^. Although similar pulses are used for irreversible electroporation, a significantly higher number of pulses are necessary for cell or tissue ablation.^30^

Therefore, the aim of our study was to perform electrotransfer of control pDNA into B16.F10 melanoma spheroids and to follow spheroid growth, transfection efficiency, expression of cytosolic DNA sensors and cytokines, and consequent cell death mechanisms.

## Materials and Methods

### Cell line and plasmid DNA

Murine melanoma cell line B16.F10 (American Type Culture Collection, Manassas, Virginia) was cultured in advanced minimum essential media (AMEM, Gibco, Thermo Fisher Scientific, Waltham, Massachusetts) supplemented with 5% fetal bovine serum (FBS; Gibco), 10 mL/L l-glutamine (GlutaMAX; Gibco), 100 U/mL penicillin (Grünenthal, Aachen, Germany), and 50 µg/mL gentamicin (Krka, Novo mesto, SIovenia) in a 5% CO_2_-humidified incubator at 37°C.

Plasmid DNA encoding enhanced green fluorescent protein, pEGFP-N1 (pEGFP, BD Biosciences Clontech, Palo Alto, California), was used for the transfection efficiency experiment and pVAX (pVAX1, Thermo Fisher Scientific) for all other experiments, both in concentration of 2 mg/mL. Amplification of pEGFP was performed in *Escherichia coli* and purified using EndoFree Plasmid Mega Kits (Qiagen, Hilden, Germany) according to the manufacturer’s protocol. The quality and quantity of isolated plasmid DNA were determined by spectrophotometric measurements of A260–A280 ratio (Epoch Microplate Spectrophotometer, Take3 Micro-Volume Plate; BioTek, Winooski, Vermont) and agarose gel electrophoresis (Scie-Plas ltd, Cambridge, United Kingdom). Plasmid pVAX was manufactured by Aldevron (Fargo, North Dakota).

### Spheroids Preparation

Melanoma cells were harvested, counted, and 300 cells were plated in each well of 96-well U-bottom plates (Corning Incorporated, Corning, New York) in 150 µL of media supplemented with hydroxypropyl methylcellulose in a final concentration of 10% (METHOCEL E50 Premium LV Hypromellose, Dow Chemical Company, Midland, Michigan). Plates were centrifuged for 2 minutes at 1000 rpm. Then, the spheroids were incubated for 3 days in a 5% CO_2_-humidified incubator at 37°C, when they reached the size of approximately 400 µm. At this day, all experiments were performed, and the day was assigned as day 0. In addition, control spheroids were embedded in paraffin, cut, and stained with hematoxylin and eosin (H&E) to visualize the presence of necrosis in untreated samples.

### Electrotransfer Protocol

First, each spheroid was transferred to a sterile 10-cm Petri dish using a pipette with wide tip opening (Thermo Fisher Scientific) to prevent damage of the spheroid. The media around spheroid was removed, the spheroid was washed with electroporation buffer (125 mmol/L sucrose, 10 mmol/L K_2_HPO_4_, 2.5 mmol/L KH_2_PO_4_, 2 mmol/L MgCl_2_ × 6H_2_0), and then 45 µL of electroporation (EP) buffer was added to all the groups. Five microliter of pDNA in concentration of 2 mg/mL of physiological saline (final amount of pDNA to each spheroid was 10 µg) was added to the plasmid groups (pEGFP or pDNA only (pDNA), pEGFP + EP, or pDNA + EP), whereas in control (Ctrl) and EP groups, 5 µL of physiological saline was added. Electrodes with 2-mm gap were placed around the spheroid, and 6 1300 V/cm pulses of 100-microsecond duration at the frequency of 4 Hz were applied with an Electro Cell B10 electric pulse generator (LEROY biotech, L’Union, France).^11^ After 5 minutes, the spheroids were transferred to 96-well U-bottom plates in cell medium containing 10% hydroxypropyl methylcellulose for further analysis (Table 1).

**Table 1.** Detailed Scheme of Performed Experiments.

Group Characteristics	Transfection	Spheroid Growth	Cell Death	qPCR
Spheroid + 45 µL of EP buffer				
Ctrl	5 µL saline	5 µl saline	5 µL saline	5 µL saline
pDNA	5 µL pEGFP	5 µL pVax	5 µL pVax	5 µL pVax
EP	5 µL saline + electroporation	5 µL saline + electroporation	5 µL saline + electroporation	5 µL saline + electroporation
pDNA+ EP	5 µL pEGFP + electroporation	5 µL pVax + electroporation	5 µL pVax + electroporation	5 µL pVax + electroporation
No. of spheroids for 1 sample	1	1	1	12
No of parallels in each experiment	2 (Ctrl, EP, pEGFP) 5 (pEGFP + EP)	12	6	2
No of experiment replication	3	2	2	2
Treatment type = electroporation	1300 V/cm, 6 pulses, 4 Hz, 100 µs (only in groups with EP)			

Abbreviation: qPCR, quantitative polymerase chain reaction.

### Permeabilization of Spheroids

Spheroid electropermeabilization was measured by propidium iodide (PI) uptake. Spheroids were prepared for electroporation as described earlier. Diluted PI (Sigma-Aldrich, St. Louis, Missouri ) was also prepared: 10 µL of 100 µmol/L PI was added to 90 µL of saline.

Two subsets of experiments were then performed. First, 5 µL of PI were added at the time of electroporation and in the other 2 hours after the electroporation. One minute after the addition of PI, images were captured at 10× objective magnification with an Olympus IX-70 (Olympus, Hamburg, Germany) and appropriate filters (excitation: 538 nm, emission: 617 nm).

### Transfection Efficiency

To determine the transfection efficiency, 24 hours after electrotransfer of pEGFP, spheroids were first imaged by fluorescence microscopy by capturing under the visible then fluorescent light, with a light exposure time of 400 milliseconds. Images were captured at 10× objective magnification with an Olympus IX-70 (Olympus) and appropriate filters (excitation: 460-490 nm, emission: 505 nm).

The same samples were then analyzed by flow cytometry. For flow cytometry analysis, spheroids were first broken into single cell suspension by adding 100 µL of trypsin and mild pipetting. Medium was added, cells were centrifuged, and resuspended in 300 µL of phosphate-buffered saline (PBS; Gibco). Cells were analyzed with FACSCanto II flow cytometer (BD Biosciences, San Jose, California), where a 488-nm laser (air-cooled, 20 mW solid state) and 530/30-nm band-pass filter were used for the excitation and detection of GFP fluorescence, respectively.

Separate samples were prepared for microscopy analysis with a ZEISS LSM 800 confocal laser scanning microscope equipped with a W Plan-Apochromat 20× 1.0 DIC (UV) VIS-IR (Carl Zeiss AG, Oberkochen, Germany) objective using a 519 nm laser, and tiles with z-stack settings were set to scan the whole spheroid in 3 dimensions to a depth of 80 µm.

Each experiment was repeated 3 times in 2 (Ctrl, EP, pEGFP) or 5 (pEGFP + EP) parallels.

### Spheroid Growth Observation

At day 0 (the day of the delivery) and every second day up to day 11, images of the spheroids were captured at 10× objective magnification with Olympus IX-70 microscope (Olympus). The area of each spheroid was measured with Fiji software.^31^ For each experimental group, 12 spheroids were measured, and average area was calculated. The experiment was repeated twice. The area of each spheroid was first normalized to day 0 and then for each group, the average was calculated and the growth curve was plotted.

### Determination of Cell Death Mechanism

Cell death mechanisms were determined by FITC Annexin V Apoptosis Detection Kit with 7-Aminoactinomycin D (7AAD) (BioLegend, San Diego, California) according to manufacturer’s instructions for flow cytometric analysis 24 hours after electrotransfer of pDNA. To obtain enough cells for the analysis, 6 spheroids were collected from 1 experimental group. The experiment was repeated twice. Spheroids were broken into single cell suspensions as described earlier. Apoptosis was evaluated by phosphatidylserine detection in the outer plasma membrane leaflet using Annexin V and necrosis with 7AAD, which has a high DNA-binding constant and can pass into the nucleus and bind to DNA in necrotic cells.

### Extraction of RNA From Spheroids and Detection of DNA Sensors and Cytokines by Quantitative Polymerase Chain Reaction

Four and 24 hours hours after electrotransfer of pDNA, spheroids were collected, and total RNA was extracted by Total RNA Kit, peqGOLD (VWR, Radnor, Pennsylvania) according to the manufacturer’s instructions. As many as 12 spheroids were collected for each experimental group to obtain enough cells to gain a sufficient amount of RNA for these experiments. The experiment was repeated twice in 2 parallels.

The concentration and purity of RNA were determined spectrophotometrically (Epoch, Biotek, Winooski, Vermont) by measurements of absorbance at 260 nm and of the ratio of absorbance at 260 and 280 nm, respectively. Reverse transcription of 500 ng of total RNA into complementary DNA (cDNA) was performed according to the manufacturer’s instructions (SuperScript VILO cDNA Synthesis Kit, Thermo Fisher Scientific). Quantitative Polymerase Chain Reaction was performed using SYBR Select Master Mix (Thermo Fisher Scientific) and custom primers for Cyclic GMP-AMP Synthase (cGAS), DDX60, DAI/ZBP1, p204, IFN-β, and TNF-α (Integrated DNA Technologies, Coralville, Iowa; Table 2) using a QuantStudio 3 Real-Time PCR System (Thermo Fisher Scientific).^11^ Relative quantification (ΔΔCt method) was performed using β-actin and glyceraldehyde 3-phosphate dehydrogenase as housekeeping genes.^32^ pDNA+EP samples were normalized to EP group. Due to the use of 12 spheroids to obtain enough material, the data are not representing the data of 1 sample but rather the average of 12 samples.

**Table 2.** Oligonucleotide Sequences for qPCR Detection of mRNA.

qPCR oligonucleotides		
DDX60	mDDX60-4160F	ACTGGAACACTCGCTTTGG
	mDDX60-4306R	GAAGTAGACATCACCCAACAGG
GAS	mcGas-for	GTGAGGACCAATCTAAGACGAG
	mcGas-rev	AGCATGTTTTCTCTATCCCGTG
DAI/ZBP1	mDAI-1081F	TGCTTTCTAGAGGACGCCACCATT
	mDAI-1213R	TGGCTTCAGAGCTTGTACCTGTGT
p204	mp204-693F	CCAGTCACCAATACTCCACAG
	mp204-831R	GAGCACCATCACTGTCAGG
IFNβ-1	IFNb1-241F	TGCCATCCAAGAGATGCTCCAGAA
	IFNb1-364R	AGAAACACTGTCTGCTGGTGGAGT
TNF-α	TNFαF	CCCTCCAGAAAAGACACCATG
	TNFαR	GTCTGGGCCATAGAACTGATG

Abbreviations: GAS, GMP-AMP synthase; DNA-dependent activator of IFN-regulatory factors; IFN-β, interferon β; qPCR, quantitative polymerase chain reaction; mRNA, messenger RNA; TNF-α, tumor necrosis factor α.

### Statistical Analysis

The data were first tested for normality of distribution with the Shapiro-Wilk test. The differences between experimental groups were statistically evaluated by one-way analysis of variance (one-way ANOVA) followed by a Holm-Sidak test for multiple comparison. A *P* value of less than 0.05 was considered as statistically significant difference. For statistical analysis and graphical representation, SigmaPlot Software (Systat Software, Chicago, Illinois) was used.

## Results

### Permeabilization of Spheroids

**Figure 1.**
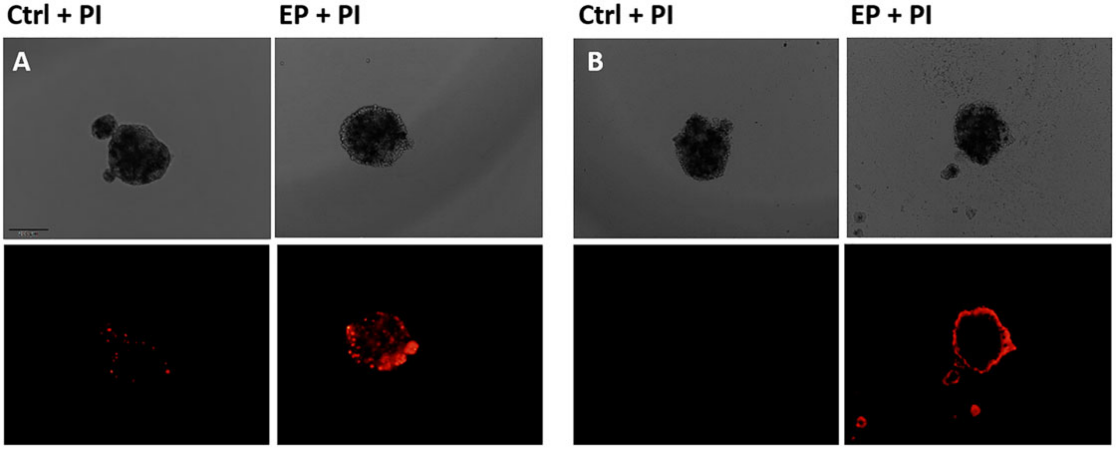
Permeabilization of spheroids 1 minute (A) and 2 hours after electroporation (EP) (B) with propidium iodide (PI). Bright-field (upper) and fluorescent (lower) images are shown. Scale Bar = 200 µm.

To determine the effectiveness of permeabilization using this pulse protocol, we added PI to the spheroids and evaluated the fluorescence under the microscope (Figure 1). One minute after electroporation, a scattered fluorescence was visible throughout the spheroid indicating an effective permeabilization (Figure 1A). To determine the resealing of the cells’ membrane after the electroporation, PI was added to the spheroids also 2 hours after the treatment. A blurry fluorescence rim was visible, indicating that certain percentage of cells died after electroporation alone (Figure 1B).

### Transfection Efficiency

Twenty-four hours (day 1) after electrotransfer of pEGFP into the spheroids, the transfection efficiency was visualized by fluorescent and confocal microscope and quantitatively determined by flow cytometry.

**Figure 2.**
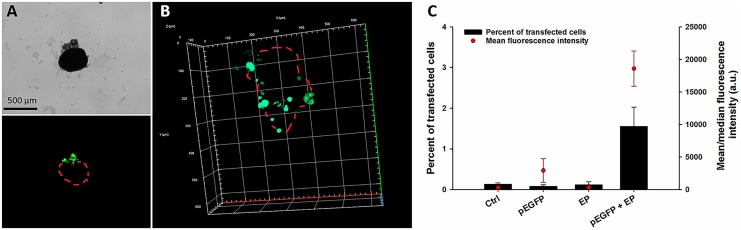
Transfection efficiency 24 hours after electrotransfer of plasmid DNA (pDNA) to spheroids with Plasmid DNA encoding enhanced green fluorescent protein (pEGFP) determined by (A) fluorescence and (B) confocal microscope and (C) by flow cytometry. Red Dots (Panel A and B) indicate approximate borders of the spheroid. *Statistically significant difference compared to all other groups (*P* < .05).

Fluorescent images showed that only the rim of each spheroid was transfected, while the core was not fluorescent 24 hours after the transfection (Figure 2A). This observation was confirmed by confocal microscopy, where also only the rim of the spheroid was transfected (Figure 2B). Probably only the part of the rim at the cathode site was transfected, since during the electroporation, pDNA is entering at cathode site and travels toward anode. Flow cytometry analysis showed <2% of transfected cells in the entire spheroid, with a median transfection efficiency ∼ 2500 a.u (Figure 1C).

### Spheroid Growth

**Figure 3.**
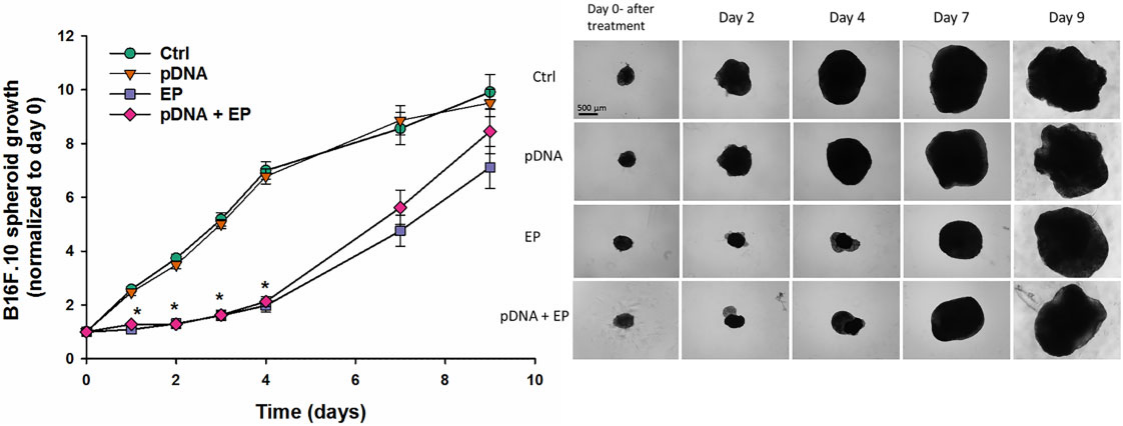
Spheroid growth after electrotransfer of pDNA. *Statistically significant difference compared to Ctrl and pDNA groups (*P* < .05).

Spheroid growth was followed up to 9 days after electrotransfer. After EP only and pDNA + EP, growth of all spheroids was delayed until day 4. Thereafter, the growth of the spheroids had the same rate as the growth of spheroids from control and plasmid-only group during exponential growth phase. The spheroids from control and plasmid groups grew exponentially and reached a plateau at day 4. By days 7 and 9, no groups differed significantly in size (Figure 3). These results indicate that after 4 days of delayed growth, spheroid growth recovered completely. These results also indicate that pDNA had no effect on spheroid growth; the delayed growth was due to the electric field application, which is in accordance with the PI data obtained 2 hours after the treatment.

### Cell Death Mechanisms

**Figure 4.**
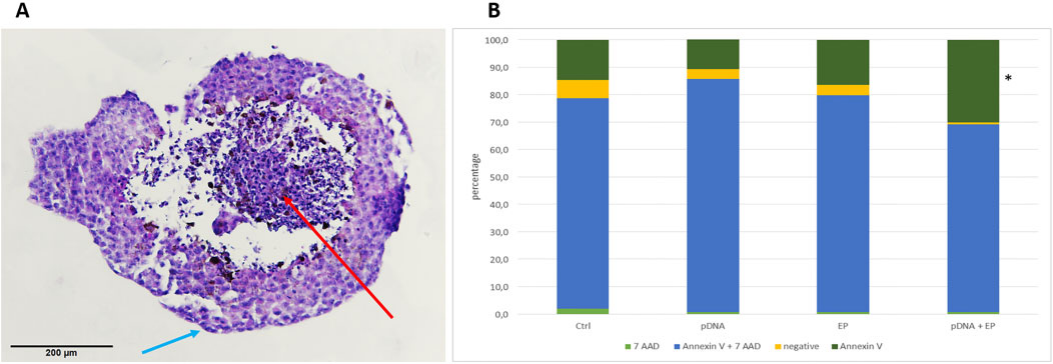
Histological image of B16.F10 spheroid stained with hematoxylin and eosin (H&E). Red arrow indicate necrotic center and blue arrows indicate cells in mitosis (A). Percentage of viable cells (negative), apoptotic (annexin V), Late apoptotic/necrotic (Annexin V + 7 AAD) and necrotic (7 AAD) cells after electrotransfer of pDNA of B16.F10 Spheroids. *P < .05 compared to control group (B).

The application of electric pulses to the spheroid can cause cell death, which may contribute to the growth delay observed in EP and pDNA+EP conditions. Therefore, a flow cytometry assay (FITC Annexin V Apoptosis Detection assay with 7AAD) was performed 1 day after the treatment to elucidate the cell death type (Figure 4). In addition, the H&E staining was done in control spheroids at the same time point. In contrast to monolayer cells, where the control sample contains approximately 97% viable cells,^11^ the percentage of viable cells in spheroids was <10% as determined by flow cytometry. Hematoxylin and eosin staining of control untreated spheroids demonstrated the necrotic center and a rim of viable cells (Figure 4A). Flow cytometry assay showed that approximately 70% to 80% of each spheroid was composed of necrotic cells (Figure 4B). After electrotransfer of pDNA, the percentage of viable (negative) cells decreased and consequently the percentage of apoptotic cells increased (Annexin V; *P* < .05). A similar pattern was previously observed after electrotransfer of pDNA of melanoma cells with identical pulses but different pDNA.^11^

### Expression of DNA Sensors and Cytokines

**Figure 5.**
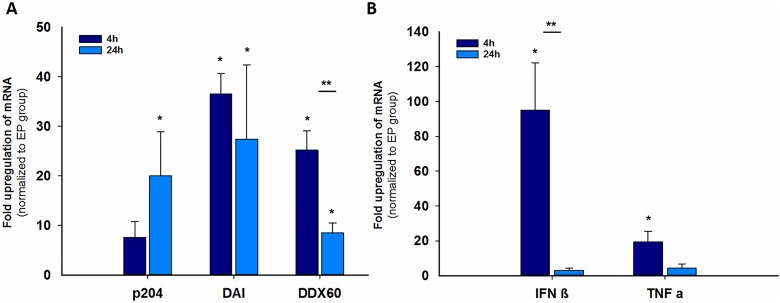
Fold upregulation of messenger RNA (mRNA) for DNA sensors (A) and cytokines (B) in spheroids 4 hours and 24 hours after electrotransfer of plasmid DNA (pDNA). *Statistically significant difference compared to control (untreated cells) group (*P* < .05), **Statistically significant difference between groups (*P* < .05).

In 2-D cultures, the messenger RNA (mRNAs) and proteins of select DNA sensors were upregulated after pDNA electrotransfer.^11^ Messenger RNA expression of different DNA sensors and cytokines was measured by qPCR. We tested the effect of pDNA electrotransfer on the mRNA levels of this subset of sensors along with a sensor for which the mRNA levels did not change, cGas, in 3-D cultures. Four hours after electrotransfer of pDNA to spheroids, statistically significantly increased levels of mRNA were detected for DAI/ZBP1 (40-fold) and DDX60 (25-fold) but not for p204 (8-fold; Figure 5). At 24 hours, the mRNA levels for those 2 sensors were reduced but still significantly upregulated compared to the EP group. On the other hand, the mRNA levels for p204 (20-fold) became significantly increased after 24 hours. No increase in cGAS mRNA levels was detected. At 4 hours, the mRNA expression significantly increased 100-fold for IFN-β and 20-fold for TNF-α compared to the EP group, while at 24 hours, the increase was no longer significant.

## Discussion

In this study, we demonstrated that electrotransfer of pDNA of tumor cells in spheroids caused increased expression of cytosolic DNA sensors and cytokines. This confirmed our results obtained in 2-D cell cultures.^11,33^

Our previous *in vitro* and *in vivo* research on B16.F10 tumor cells and murine tumor models demonstrated that after electrotransfer of pDNA, there is a pronounced effect on the growth of tumor cells and on experimental tumors. *In vitro*, these effects correlated with changes in the expression of a subset of DNA sensors.^11^ The same observations were made in the present study on a spheroid model of B16.F10 cells where these same DNA sensors were upregulated. The peak upregulation was detected 4 hours after electrotransfer of pDNA and decreased over time. The upregulation of DDX60 and p204 DNA sensors was very similar to the upregulation obtained in 2-D cell culture; however, the upregulation of DAI/ZBP1 was significantly lower (*P* < .05).

The levels of IFN-β levels were significantly higher (*P* < .05) to that obtained in 2-D cell cultures but much higher than the levels obtained in tumors.^11^ Interestingly, in melanoma tumors *in vivo,* DNA sensor mRNAs were not upregulated, whereas IFN-β levels were significantly increased on mRNA and protein level.^11^ This indicates that the presence of other cells in tumors, particularly immune cells, may complicate the detection of DNA sensor upregulation while contributing to the production of cytokines. Since upregulation is not synonymous with activation, it is also possible that alternative sensors may be initially activated and responsible for the production of INF-β. The specific upregulated sensors may act later in the pathway.

The transfection efficiency of electrotransfer using a plasmid-encoding GFP was very low and comparable to that obtained *in vivo*. The transfection pattern was very similar as in *in vivo* models; mainly the rim was transfected while the core remained intact.^34^ Thus from this point of view, spheroids represent a good model for tumors *in vivo*. Furthermore, the very low transfection efficiencies confirmed results obtained in other studies using different cell types or spheroids composed of 2 cell types.^35,36^

Although the control spheroids contain a very high percentage of necrotic cells, the increase in percentage of apoptotic cells in spheroids after electrotransfer was detectable. The electrotransfer of pDNA caused similar pattern of cell death as was previously observed in 2-D cell cultures.^11^ Specifically, an increased proportion of apoptotic cells was detected without additional necrotic cells. Despite the significant loss of cell viability in pDNA+EP group compared to other groups, the growth rate of the spheroids treated with EP alone and combined with pDNA was identical. This growth was delayed until day 4 and then accelerated to achieve the same size as control spheroids by day 7. This effect of EP alone was previously observed in other studies and was ascribed to the loss of viability of the cells in outer layers.^37,38^ The loss of cell viability was ascribed to the cell size, the size of necrotic core, and other cell properties within the spheroids, such as cell-to-cell junctions and extracellular matrix secretion.^37,38^ However, such viability loss is not in accordance with the results obtained either in 2-D cell cultures or in tumors, where the addition of pDNA to EP significantly reduced cell viability. In tumors, an additional difference in cell survival could be explained by the activation of immune system. However, the difference between 2-D cultures and spheroids is difficult to explain and deserves further studies.^14^

In conclusion, our results on spheroids confirm the results obtained in 2-D cell cultures demonstrating that electrotransfer of pDNA to tumor cells in spheroids upregulated cytosolic DNA sensors and cytokines. In addition, the growth of spheroids was delayed after electric field application.

## Abbreviations

cGAS: cyclic GMP-AMP synthase
DAI/ZBP1: DNA-dependent activator of IFN-regulatory factors
DDX60: DEAD (Asp-Glu-Ala-Asp) Box Polypeptide 60
EP: electroporation
IFN-β: interferon β
p204: interferon-activated gene 204
PI: propidium iodide
pDNA: plasmid DNA
TNF-α: tumor necrosis factor α.
